# The effect of omega-3 supplementation on androgen profile and menstrual status in women with polycystic ovary syndrome: A randomized clinical trial

**Published:** 2013-08

**Authors:** Azadeh Nadjarzadeh, Razieh Dehghani Firouzabadi, Niloofar Vaziri, Hoorieh Daneshbodi, Mohammad Hassan Lotfi, Hassan Mozaffari-Khosravi

**Affiliations:** 1 *Department of Nutrition, Faculty of Health, Shahid Sadoughi University of Medical Sciences, Yazd, Iran.*; 2 *Research and Clinical Center for Infertility, Shahid Sadoughi University of Medical Sciences, Yazd, Iran.*; 3 *International Campus of Shahid Sadoughi University of Medical Sciences, Yazd, Iran.*; 4 *Department of Biostatistics and Epidemiology, Faculty of Health, Shahid Sadoughi University of Medical Sciences, Yazd, Iran.*

**Keywords:** *Polycystic ovary syndrom*, *Sex hormone*, *Binding globulin*, *Omega-3*

## Abstract

**Background:** There is some evidence regarding the effect of poly unsaturated fatty acid intake on androgen levels and gonadal function in polycystic ovary syndrome (PCOS).

**Objective:** This study was conducted to determine the effect of omega-3 supplementation on sex hormone-binding protein (SHBG), testosterone, free androgen index (FAI) and menstrual status in women with PCOS.

**Materials and Methods:** This double-blind randomized clinical trial was conducted on 78 overweight/obese women with PCOS. Participants were randomized to receive omega-3 (3gr/day) or placebo for 8 weeks. Data about weight, height and nutrient intake as well as blood samples were collected before and after intervention. Serum concentrations of testosterone (nmol/L) and SHBG (nmol/L) were measured. FAI was also calculated as the ratio of testosterone to SHBG.

**Results:** Seventy eight patients (age: 26.92±5.46 yrs, Body Mass Index: 31.69±4.84 Kg/m^2^) completed the study. There was no significant difference in mean age, weight, height, Body Mass Index and intake of energy, and macronutrients between 2 study groups before and after treatment. All the participants had irregular periods. After the trial the percentage of regular menstruation in the omega-3 group was more than the placebo group (47.2% vs. 22.9%, p=0.049). Furthermore, testosterone concentration was significantly lower in the omega-3 group compared with placebo, after supplementation (p=0.04). SHBG and FAI did not change in either group.

**Conclusion:** Omega-3 supplementation could reduce serum concentrations of testosterone and regulate menstrual cycle without significant effect on SHBG and FAI. Future studies with longer period of supplementation are warranted.

This article extracted from M.Sc. thesis. (Niloufar Vaziri)

**Registretion ID in IRCT:** IRCT201112318564N1

## Introduction

Polycystic ovary syndrome (PCOS) is the commonest endocrine disorder and one woman out of every fifteen suffers from this disorder in the world (1). In 2003 the European Fertility, Embryology Association and the American Fertility Association described the diagnostic criteria of PCOS in Rotterdam conference. It will confirm the existence of this disease if 2 of the following criteria exist. The diagnostic criteria are oligoovulation or anovulation, the clinical symptom of hyperandrogenism or the presence of hyperandrogenemia, the view of polycystic ovaries in ultrasound (2, 3). 5-10% of women in fertility ages are influenced by this syndrome which is considered the most usual endocrine disorder in women in the USA (1, 4). 

Aziz and colleagues' study on 400 women has shown that prevalence of PCOS is growing and the exact cause is not known (4, 5). Nowadays, it is proved that this syndrome is due to genetic and environmental factors. Insulin resistance and hyperinsulinemia are the aggravating factors in the incidence of this problem (6, 7). 20-40% patients with PCOS have insulin resistance. This condition is most commonly seen in obese ones (8, 9). This syndrome is associated with infertility, type 2 diabetes, lipid and lipoprotein disorders, mild chronic inflammation, and cardiovascular disease (10). The primary studies have shown that disorders in menstrual status in polycystic ovary syndrome are due to the decrease of sex hormone binding protein levels and the increase of androgen levels in serum and plasma (11-14). 

Omega-3 fatty acids, which have positive effects on health, are the precursors of eicosanoids. Most studies in the past ten years have shown the positive relationship between omega-3 fatty acids intake and androgen levels (15, 16). Hyperandrogenism as the first symptom in PCOS is due to irregularity in steroid production (3). This condition can be caused by an increase in estrogen secretion (17). LH hypersecretion can lead to increase in serum levels 17 hydroxy progesterone, testosterone, and androstendion (18). 

The question, whether or not omega-3 supplements can change androgen levels despite the hormone imbalances in them, has not been answered. In addition, studies are very limited on this field. We know that infertility has the negative psychological and social effects and the treatment of infertility is a high cost process. As the prevalence of PCOS was higher in women with overweight, so we investigated the effect of omega-3 in overweight and obese PCOS affected women (8, 9). We aim to investigate effect of omega-3 as a food supplement for aiding the treatment of women with PCOS. So, this study was performed to investigate the effect of omega-3 on sex hormone binding protein (SHBG), testosterone, free androgen index and menstrual status in women with polycystic ovary syndrome.

## Materials and methods

This study was a randomized double blind placebo controlled clinical trial that was executed from November 2011 to May 2012 among the women with PCOS (according to Rotterdam criteria) referred to the Research and Clinical Center for Infertility and Shahid Sadoughi Hospital, Yazd, Iran. This study was supported by Yazd Research and Clinical Center for Infertility. 

35 women for each group were estimated. Then we added 20% to this number, so 39 people for each group were included in the analyses. Individuals were eligible for participation if they were aged 20-40 years, have body mass index of over 25, and met the Rotterdam criteria for PCOS. The PCOS diagnostic criteria were oligoovulation or anovulation, the clinical symptom of hyperandrogenism or the presence of hyperandrogenemia, and diagnosis of polycystic ovaries using ultrasound. The women who were menopause, consumed omega-3 in the last 3 months, used tobaccos, were diabetic or hypothyroidism, followed a special diet or consumed effective drugs on hormonal profile like oral conceptives (OCP), glucocorticoids, ovulation induction agents, anti-obesity, antidiabetic, antihypertensive medicine, estrogenic, antiandrogenic, anticoagulants and antidepressants, in the last 3 months before enrollment were excluded. If patients need treatment with OCP, they were excluded from the study. Because of difference in hormone profile status between menopause and menstruation, menopausal women excluded from the study.

At first, demographic questionnaire was completed for all participants. We completed 24 hr dietary recall for each subject before and after intervention to eliminate the probable effect of change in dietary intake of macro and micronutrients. The data of recalls were analyzed by nutrition software, Nutritionist 4 (N-Squared Inc, San Bruno, CA, USA). Height and weight were measured by Seca scale and stadiometer with accuracy 0.1 Kg and 0.5 Cm, respectively. Participants were randomly assigned into two groups using a random number table. 

Placebo and omega-3 were in the same form of package and the patients and researcher weren't aware of the content of the pack until the end of analysis. Subjects were randomized, in a 1-1 ratio, to receive either omega-3 or placebo (paraffin) both were purchased from Zahravi pharmaceutical Company (Tabriz, Iran). Patients were asked to take three capsules every day up to 8 weeks (19, 20). An adherence to the study was monitored by capsule counting and self-report. If they consumed less than 80% of the prescribed supplements, they were excluded from the analysis. Subjects were visited at 2 week intervals to assess safety and compliance. They were requested not to change their usual diet, physical activity and to avoid taking any other dietary supplements during the study. This study was approved by Shahid Sadoughi University of Medical Sciences Ethics Committee and written informed consent was obtained from all participants. 

**Biochemical and hormonal assessment**

10^cc^ of venous blood was collected before and after the trial. Serum samples were stored in -80^o^C for later analysis. The serum concentration level of testosterone and SHBG were measured using direct immunoenzymatic assay (DiaMetra, Italy) and Elisa Reader (Statfax 2600, USA). Free androgen index was also calculated as the ratio of testosterone to SHBG. 

**Statistical analysis**

Data were analyzed and reported only for patients who completed the trial. Statistical analysis of data was performed using SPSS version 12 software (SPSS Inc, Chicago IL. Version12). To compare qualitative variables between groups Chi-square test was performed. The normal distribution of all studied parameters was checked with Kolmogorov-Smirnov test. Student t-test and paired t-test were used for variables which were distributed in a normal way, besides Mann-Whitney and Wilcoxon test were performed for variables that have not normal distribution. The two tailed p-value less than 0.05 were considered significant.

## Results

**Figure 1:**
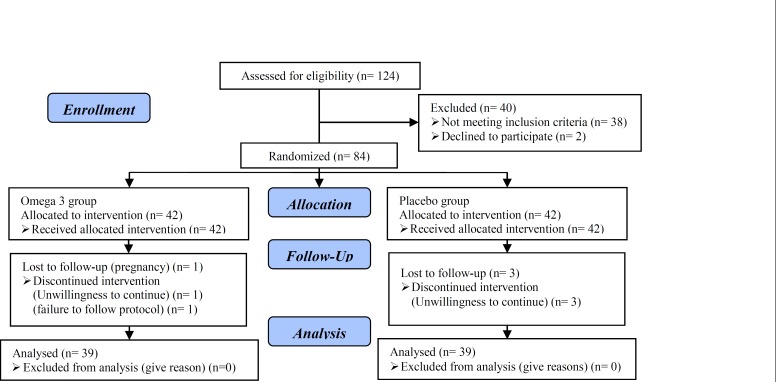
Consort flow diagram

78 (92.8%) participants completed the study. Six persons (3 in the placebo and 3 in the omega-3 group) withdrew from the study because of personal reasons, pregnancy and failure to follow the protocol (Figure 1). The mean age of the patients who finished the study in 2 groups was 26.92±5.46 years old. No significant statistical difference between groups was found at baseline in terms of education, job situation, adherence to exercise program, and marital status. In addition at the beginning of the study, there was not significant statistical difference between the groups in term of age. 

Mean weight, height, and body mass index (BMI) were 79.52 Kg, 158.41 cm, and 31.69 Kg/m^2^, respectively (Table I). Student *t *- test between the studied groups showed no significant statistical difference in terms of mean age, weight, and BMI. There was no significant statistical difference in terms of daily dietary energy, carbohydrate, protein, and fat between the studied groups at the beginning and the end of the study (Table II). Baseline mean testosterone concentrations between the two groups receiving omega-3 and placebo had no significant statistical difference. 

However mean concentration of this hormone was significantly different between two groups (p=0.04). As shown in table III, changes in mean concentration of testosterone between the two studied groups weren't statistically significant. Mean concentration of SHBG between the two groups receiving omega-3 and placebo showed no significant statistical difference before the trial (p=0.87).There wasn't any noticeable statistical difference between the two studied groups (Table III). 

Since the distribution was not normal in free androgen index variable, Wilcoxon test was performed. The results of this test showed that in the group receiving omega-3 median of FAI decreased by 0.09 after 8 weeks. However, the decrease was not significant (p=0.08). According to Mann-Whitney test results, although there was a change in the median value after 8weeks, it wasn't statistically significant between the two studied groups (p=0.17) (Table IV). All the patients entering the study had irregular periods. But, after the trial, the percentage of regular menstruation in the group receiving omega-3 was more than the placebo group (p=0.049) (47.2% vs. 22.9) (Table V).

**Table I:** Mean of age and anthropometric variables in omega-3 and placebo groups at the baseline

**Variables**	**Placebo (n=39)**	**Omega-3 (n=39)**	**p-value** *
Weight (Kg)	15.61 ± 79.11	11.23 ± 79.94	0.78
Height (cm)	5.81 ± 158.46	7.74 ± 158.35	0.94
BMI (Kg/m^2^)	5.74 ± 31.46	3.86 ± 31.88	0.7
Age (year)	5.91 ± 26.91	5.05 ± 26.92	0.99

Data are presented as mean±SD.

* Independent Student t-test.

**Table II:** The daily dietary intakes of energy and macronutrients in omega-3 and placebo groups before and after supplementation

**Daily dietary intake**		**Placebo (n=39)**	**Omega-3 (n=39)**	**p-value** **
Energy (Kcal)				
	Before	262.14 ±2020.2	235.36 ± 1970	0.49
	After	213.63 ± 1998.5	223.89 ± 1960.6	0.57
	p-value*	0.32	0.63	
Carbohydrate (g)				
	Before	58.6 ± 268.61	32.3 ± 260.21	0.57
	After	41.28 ± 266.55	29.17 ± 259.21	0.36
	p-value*	0.91	0.65	
Fat (g)				
	Before	19.15 ± 78.42	19.23 ± 79.03	0.85
	After	20.20 ± 76.56	20.25 ± 79.47	0.52
	p-value*	0.52	0.51	
Protein (g)				
	Before	11.39 ± 63.68	10.20 ± 60.03	0.14
	After	12.83 ± 65.15	10.38 ± 59.64	0.09
	p-value*	0.30	0.80	

Data are presented as mean±SD.

* Paired sample t-test.

** Independent Student t-test.

**Table III:** The mean and mean of change of testosterone and SHBG concentration in omega-3 and placebo groups before and after supplementation

**Group**		**Placebo (n=39)**	**Omega-3 (n=39)**	**p-value** **
Testosterone (ng/ml)				
	Before	0.39 ± 0.65	0.22 ± 0.54	0.13
	After	0.3 ± 0.63	0.25 ±0.5	0.04
	p-value*	0.7	0.1	
	Changes(ng/ml)	0.29 ± -0.01	0.14 ± -0.03	0.69
SHBG (nmol/L)				
	Before	21.3 ± 35.41	20.18 ± 36.18	0.87
	After	23.3 ± 36.85	21.53 ± 38.02	0.81
	p-value*	0.28	0.18	
	Changes(nmol/L)	8.28 ±1.44	8.56 ± 1.84	0.83

Data are presented as mean±SD.

* Paired sample Student t-test.

** Independent Student t-test.

**Table IV:** The Mean, mean difference, median, of free androgen index in both groups before and after supplementation

**Placebo (n=39)**	**Mean** **±** **SD**	**median**	**Omega-3 (n=39)**	**Mean** **±** **SD**	**Median**	**p-value** **
Before	3.38 ± 2.94	1.47	Before	1.74 ± 2.18	1.79	0.17
After	2.87 ± 2.83	1.78	After	1.62 ± 1.95	1.7	
p-value*	0.95		p-value*	0.08		
Changes	1.79 ± -0.1	0.00	Changes	0.77 ± -0.22	-0.13	

* Wilcoxon test

** Mann-Whitney test

**Table V:** Menstrual status in omega-3 and placebo groups at the end of study

**Menstrual status**	**Placebo (n=39)**	**Omega-3 (n=39)**	**p-value** *
Regular [n (%)]	8 (22.9%)	17 (47.2%)	0.049
Irregular [n (%)]	27 (77.1%)	19 (52.8%)	

* Chi-Square test

## Discussion

The results of this study show that omega-3 supplementation result in decrease in testosterone concentration after 8 weeks trial. This decrease can be as a result of the effect of omega-3 on LH. Hyperandrogenism is the first clinical symptom in these patients, and it is due to irregularity in steroid production (3). LH hypersecretion can lead to increase in serum levels 17 hydroxy progesterone, testosterone, and androstendion (18). 

In patients with PCOS, increased serine phosphorylation of the insulin receptor, resulting in activation of both ovarian and adrenal P450c17a enzymes and finally promoting androgen synthesis (21). Phelen *et al* showed that a greater plasma n-6 PUFA concentration and n-6:n-3 PUFA ratio was associated with higher circulating androgens and that plasma LC n-3 PUFA status was associated with a less atherogenic lipid profile. LC n-3 PUFA supplementation reduced plasma bioavailable testosterone concentrations. So, PUFA consumption could change androgen profile (22). 

Also Akinsete *et al* noted that omega-3 intake could decrease possibility of the risk of prostate cancer in rat by decreasing of testosterone concentration (23). Our findings are different from Kasim-Karakas *et al* research conducted on 17 PCOS women (12). It seems that different results are because of small sample size and not having placebo group. The present study shows that mean and changes in mean value in SHBG after trial in the two studied groups were not statistically significant. 

It may indicate that the more duration of study, the more significant the levels of this variable in the study. This finding is not unexpected because insulin resistance is one of the clinical symptoms of this disease. Since there is an opposite association between SHBG serum levels and insulin, the presence of hyperinsulinemia in these people can inhibit hepatic synthesis of SHBG. Similar to our findings, Kasim-Karakas et al reported that mean concentration of sex hormone binding protein wasn't statistically significant after having a diet rich in poly unsaturated fatty acids (12). 

The results of some studies have shown that fasting insulin level, lipid profile and free androgen index were noticeably higher in PCOS women compared with healthy subjects. Therefore, decisions must be taken in order to reduce the risk of cardiovascular disease in these patients (24). Measuring free testosterone level and free androgen index is one of the sensitive methods to determine the increased amount of androgens. Free androgen index is calculated from the ratio of testosterone to SHBG. Our study showed that median free androgen index increased by 0.09 after 8 weeks in the group receiving omega-3, although it hasn't shown any statistically significant difference. 

This decline was the result of testosterone level decrease. Some studies have shown that omega-3 supplements compared with placebo reduce liver fat significantly, yet it doesn't have any noticeable effect on androgens (25). Moreover, Kuzmanov had similar result to our findings which has shown that 3 months supplementation of omega-3 had no significant effect on hormones (26). Disturbance of menstrual cycle is one of the features of polycystic ovary syndrome. Approximately 85-90 percent of patients experienced oligomenorrhea and about 30-40 percent experienced amenorrhea. Obesity and weight-related problems in childhood are the cause of these problems (27). 

Marshall Keri *et al* reported that PCOS women have clinical problems such as disruption of menstrual cycle, infertility, and hirsutism. They mentioned if the patients weren't treated well, they would be prone to type 2 diabetes, cardiovascular disease, and hyperstrogen-related cancer (28). All the subjects participated in our study suffer from irregular period. While after the trial, the percentage of regularity in menstrual status in the omega-3 group was significantly higher compared with the placebo group (47.2% vs. 22.9%). 

This result can be due to effects of omega-3 on testosterone concentration. We could not find any study which determines the effect of omega-3 supplements on regularity the menstrual cycle. Most of the studies mostly focus on dysmenorrhea status. The constraints on the duration of the project and sample size can be pointed out as the limitations of this project. Studies with longer period of time are suggested in order to get more effective results. It is also advisable that the effect of Docosa Hexaenoic Acid (DHA) and Eicosa Pentaenoic Acid (EPA) will be studied separately, on the mentioned variables. 

## Conclusion

In summary, 8 weeks supplementation with 3gr of omega-3 could reduce serum concentrations of testosterone in overweight and obese PCOS patients. In addition, menstrual cycle becomes more regular in this group of subjects. But it does not change the free androgen index and concentratione of sex hormone binding protein. Trials with longer duration are warranted.

## Conflict of interest

The authors declare that there is no conflict of interest in this study.
